# Microcirculatory Response to Blood vs. Crystalloid Cardioplegia During Coronary Artery Bypass Grafting With Cardiopulmonary Bypass

**DOI:** 10.3389/fmed.2021.736214

**Published:** 2022-01-13

**Authors:** Güclü Aykut, Halim Ulugöl, Uğur Aksu, Sakir Akin, Hasan Karabulut, Cem Alhan, Fevzi Toraman, Can Ince

**Affiliations:** ^1^Department of Intensive Care, Laboratory of Translational Intensive Care, Erasmus Medical Center, Erasmus University Rotterdam, Rotterdam, Netherlands; ^2^Department of Anaesthesiology and Reanimation, School of Medicine, Acibadem Mehmet Ali Aydinlar University, Istanbul, Turkey; ^3^Department of Biology, Faculty of Science, University of Istanbul, Istanbul, Turkey; ^4^Department of Intensive Care, Haga Teaching Hospital, The Hague, Netherlands; ^5^Department of Cardiovascular Surgery, School of Medicine, Acibadem Mehmet Ali Aydinlar University, Istanbul, Turkey

**Keywords:** microcirculation, cardiopulmonary bypass, systemic inflammatory response, blood cardioplegia, crystalloid cardioplegia, incident dark-field imaging

## Abstract

**Background:** Blood cardioplegia attenuates cardiopulmonary bypass (CPB)-induced systemic inflammatory response in patients undergoing cardiac surgery, which may favorably influence the microvascular system in this cohort. The aim of this study was to investigate whether blood cardioplegia would offer advantages over crystalloid cardioplegia in the preservation of microcirculation in patients undergoing coronary artery bypass grafting (CABG) with CPB.

**Methods:** In this prospective observational cohort study, 20 patients who received crystalloid (*n* = 10) or blood cardioplegia (*n* = 10) were analyzed. The microcirculatory measurements were obtained sublingually using incident dark-field imaging at five time points ranging from the induction of anesthesia (T_0_) to discontinuation of CPB (T_5_).

**Results:** In the both crystalloid [crystalloid cardioplegia group (CCG)] and blood cardioplegia [blood cardioplegia group (BCG)] groups, perfused vessel density (PVD), total vessel density (TVD), and proportion of perfused vessels (PPV) were reduced after the beginning of CPB. The observed reduction in microcirculatory parameters during CPB was only restored in patients who received blood cardioplegia and increased to baseline levels as demonstrated by the percentage changes from T_0_ to T_5_ (%Δ)_T0−*T*5_ in all the functional microcirculatory parameters [%ΔTVD_T0−*T*5_(CCG): −10.86 ± 2.323 vs. %ΔTVD_T0−*T*5_(BCG): 0.0804 ± 1.107, *p* < 0.001; %ΔPVD_T0−*T*5_(CCG): −12.91 ± 2.884 vs. %ΔPVD_T0−*T*5_(BCG): 1.528 ± 1.144, *p* < 0.001; %ΔPPV_T0−*T*5_(CCG): −2.345 ± 1.049 vs. %ΔPPV_T0−*T*5_(BCG): 1.482 ± 0.576, *p* < 0.01].

**Conclusion:** Blood cardioplegia ameliorates CPB-induced microcirculatory alterations better than crystalloid cardioplegia in patients undergoing CABG, which may reflect attenuation of the systemic inflammatory response. Future investigations are needed to identify the underlying mechanisms of the beneficial effects of blood cardioplegia on microcirculation.

## Introduction

The use of cardiopulmonary bypass (CPB) is associated with a wide range of stressors affecting the microvascular system (1). Among others, these stressors include hypotension, hemodilution, hypothermia, cardiac arrest, and non-pulsatile flow. Moreover, many factors during CPB, such as contact of the blood components with the artificial surface of the CPB circuit, ischemia-reperfusion injury (IRI) of the heart, or the release of endotoxins, have been reported to induce a systemic inflammatory response (2). This syndrome results in the activation of the innate immune system *via* inflammatory mediators, which, in turn, leads to the recruitment of leukocytes into sites of inflammation, thereby causing endothelial injury (2). As a result, microcirculatory alterations may occur, leading to inadequate tissue perfusion and oxygenation (1).

Intraoperative cardiac arrest during coronary artery bypass grafting (CABG) is induced by cardioplegia. One of the most important advantages of the use of cardioplegia in cardiac surgery is its protection of myocardial function while simultaneously enabling a stable and bloodless operative field to allow the surgical procedure. Since Melrose and colleagues introduced this method as a means for elective cardiac arrest (3), many attempts have been made to develop an optimal regime for myocardial preservation. The use of blood added to the cardioplegia solution was found to notably optimize the myocardium during the CPB-induced global hypoperfusion and to provide a protective effect on the myocardium under ischemic conditions (4). Beyond this preventive effect, blood cardioplegia appears to attenuate the CPB-induced systemic inflammatory response (5, 6), which may, in turn, affect the microcirculatory perfusion and oxygenation.

The effects of CPB on the microcirculation and the resultant microcirculatory changes during cardiac surgery have been investigated in several observational studies (7–10). Since no data are available on the precise influence of cardioplegia type on these CPB-induced microcirculatory alterations, we investigated whether blood cardioplegia would offer advantages over crystalloid cardioplegia in the preservation of microcirculation in patients undergoing CABG with CPB.

## Materials and Methods

### Study Design and Setting

This prospective observational cohort study was conducted at Acibadem Mehmet Ali Aydinlar University School of Medicine and was approved by the Ethics Committee of this institution (ATADEK 2013-540). All the procedures that were followed in this study were in accordance with the Declaration of Helsinki 1964 and its later amendments. Informed written consent was obtained from all the participants. The reporting of this study follows the recommendations of the Strengthening the Reporting of Observational Studies in Epidemiology (STROBE) statement (11).

### Enrollment of Patients

Patients scheduled for isolated coronary bypass graft surgery with CPB were consecutively enrolled during a period of 2 months. Exclusion criteria were withdrawal of consent, previous heart or oral surgery, emergency surgery, ejection fraction < 30%, pregnancy, history of myocardial infarction, systemic inflammatory disease or a history of immunosuppressive drugs or steroids, age below 18 years, or vasculitis. The study protocol did not include any intervention, as the cardioplegia type to be used was prescribed by the surgeon, irrespective of enrollment of patients. Patients planned to receive crystalloid or blood cardioplegia, were enrolled, and assigned for crystalloid [crystalloid cardioplegia group (CCG)] or blood [blood cardioplegia group (BCG)] cardioplegia group.

### Variables

Parameters of microvascular density [total vessel density (TVD) and perfused vessel density (PVD)] and microvascular perfusion [proportion of perfused vessels (PPV)] were the primary endpoints of interest. Microcirculatory measurements were performed after the induction of anesthesia (T_0_), 5 min after the heparin administration before the onset of CPB (T_1_), 5 min after the onset of CPB before the cardioplegia delivery (T_2_), during CPB 10 min after the application of the cross-clamp (T_3_), after the cross-clamp release when the heart began to beat (T_4_), and following CPB 10 min after the protamine administration (T_5_). At each time point, arterial blood gas analysis was executed and hemodynamic variables, body temperature, hematocrit, and lactate concentrations were recorded. Demographic characteristics, durations of surgery, CPB, and cross-clamp periods of all the patients were collected. With respect to the postoperative outcome, the postoperative intubation time and the durations of intensive care unit (ICU) and hospital stay were studied.

### Clinical Practice

#### Anesthesia Protocol

All the surgeries were performed under general anesthesia. Prior to anesthetic induction, arterial catheters were inserted for hemodynamic monitoring during surgery. Anesthesia was induced using fentanyl (15–25 μg/kg), propofol (1 mg/kg), and vecuronium (0.05–0.1 mg/kg) and maintained *via* continuous propofol infusion (200–400 mg/h), intermittent doses of fentanyl (1–3 μg/kg), and vecuronium (0.02–0.05 mg/kg). Patients received mechanical ventilation with tidal volumes of 6–8 ml/kg, a fraction of inspiratory oxygen (O_2_) of 45%, and a positive end-expiratory pressure of 5 cm H_2_O. Respiratory rate was adjusted to achieve end-tidal carbon dioxide (CO_2_) partial pressure values between 35 and 40 mm Hg.

#### Surgery and Extracorporeal Circulation (ECC)

The heart was exposed following median sternotomy. Arterial cannulation was performed at the ascending aorta proximal to the brachiocephalic truncus and the two-stage venous cannulation was achieved *via* the right atrial appendage. CPB was initiated after heparin administration when the activated coagulation time exceeded 480 s and was performed with a standard roller pump using an S3 heart–lung machine (Stöckert Sorin Group Deutschland GmbH, Munich, Germany) combined with a heater-cooler device (3M Sarns TCM II, Michigan, USA). The extracorporeal circuit consisted of tubing sets (Bicakcilar, Ref: 30008351, Istanbul, Turkey), a hollow fiber membrane oxygenator (Dideco Compactflo Evo Physio/M, Ref: 050516, Sorin Group, Mirandola, Italy), and an arterial line filter (Bicakcilar, Istanbul, Turkey). The priming solution for ECC included 1,090 ml of Ringer's lactate solution, 150 ml of 20% mannitol, 60 ml of 8.4% sodium bicarbonate, and 10,000 IU heparin. During CPB, moderate hypothermia (32–35°C) was provided. The mean arterial pressure (MAP) and non-pulsatile flow rates were maintained between 40 and 80 mm Hg and 2–2.5 L/min/m^2^, respectively. A hematocrit value <24% during CPB was considered as the trigger point for red blood cell (RBC) transfusion. Myocardial viability was preserved *via* crystalloid or blood cardioplegia delivered into the aortic root in an antegrade fashion. The crystalloid cardioplegia was composed of cold (4–8°C) Plegisol cardioplegic solution, available in a 1-L bag, to which 10 ml of 8.4% sodium bicarbonate was added prior to administration. To prepare the blood cardioplegia solution, oxygenated blood drawn from CPB circuit was mixed in a 3:1 ratio with a cold crystalloid solution (4–8°C), which contained 120 ml of acid citrate dextrose-A (ACD-A), 20 ml of 7.5% potassium chloride, 10 ml of 8.4% sodium bicarbonate, 10 ml of 15% magnesium sulfate, and 80 ml of 5% dextrose. The potassium concentration in the entire cardioplegic solution was 21 mEq/l. The entire cardioplegic solution was then cooled in ice, until it was given by the anesthetist *via* a pressure bag. In the both study groups, cardiac arrest was achieved by an initial volume of 700 ml cardioplegia, followed by an additional dose of 300 ml given after 15–20 min. The target administration pressure was 200–300 mm Hg and the target administration flow was 100–200 ml/min. The weaning from CPB was commenced at a rectal temperature of 37°C. When the patient was hemodynamically stable, ECC was discontinued and, finally, protamine sulfate was administered.

### Microcirculatory Measurements

Microcirculatory measurements were performed sublingually with a handheld camera based on incident dark-field (IDF) imaging (Cytocam, Braedius Medical, Huizen, The Netherlands). Cytocam-IDF imaging has been described extensively elsewhere (12). Various precautions were taken and steps followed in line with international guidelines to obtain images of adequate quality and to ensure satisfactory reproducibility (13). First, the focus and illumination were adjusted. Subsequently, three steady images of 4 s were acquired and stored on a computer in accordance with the current international guidelines on sublingual microcirculation (13). The image clips were then exported using the embedded CC-tools software (Cytocam, Braedius Medical, Huizen, The Netherlands) for offline analysis.

### Microcirculatory Image Analysis

Offline image analysis was performed in patients who had recordings with good or acceptable image quality (14). The images were analyzed to measure the microcirculatory parameters TVD, PVD, and PPV by means of software-assisted analysis (AVA 3.2; Automated Vascular Analysis, Academic Medical Center, University of Amsterdam, Amsterdam, The Netherlands) (15) in line with international consensus (13). The analysis of TVD was restricted to vessels with a diameter < 20 μm. A semiquantitative analysis enabled to distinguish among vessels with no flow (0), intermittent flow (1), sluggish flow (2), and continuous flow (3). PPV (%) was calculated as the number of vessels with flow values of 2 and 3 divided by the total number of vessels. PVD (mm/mm^2^) was determined as TVD multiplied by the fraction of perfused vessels. The analysis of all the images was performed in a blinded fashion and a randomized order according to the international guidelines (13).

### Statistical Analysis

The sample size of this study was based on previous findings of the group, showing an increase in microcirculatory density from 10.5 ± 1.2 to 12.9 ± 1.2 mm/mm^2^ following RBC transfusion in patients undergoing on-pump cardiac surgery (16). A power of 95% and an alpha of 0.05 were used in the sample size calculation. The required sample size for each group was eight. Two patients were added to each group to account for possible lack of data during the study period. Data were analyzed using the GraphPad Prism version 6 (GraphPad Software Incorporation, San Diego, California, USA) by independent researchers. All values are presented as mean ± standard error of the mean (SEM) or median with an interquartile range. The Kolmogorov–Smirnov test was used to determine whether the data were distributed normally. The repeated-measures ANOVA with *post-hoc* Tukey's test were performed to analyze the time-dependent differences within groups for microcirculatory and clinical parameters. Demographic parameters, the differences between groups at individual time points, and the percentage changes in microcirculatory parameters from T_0_ to T_5_ were analyzed *via* the unpaired *t*-test for parametric data and the Mann–Whitney *U*-test for non-parametric data. Correlations between changes in hemodynamic parameters and alterations in microcirculatory parameters during CPB were tested with the Pearson's correlation analysis. *P* < 0.05 was considered as statistically significant.

## Results

### Characteristics of Patients

**Figure 1:**
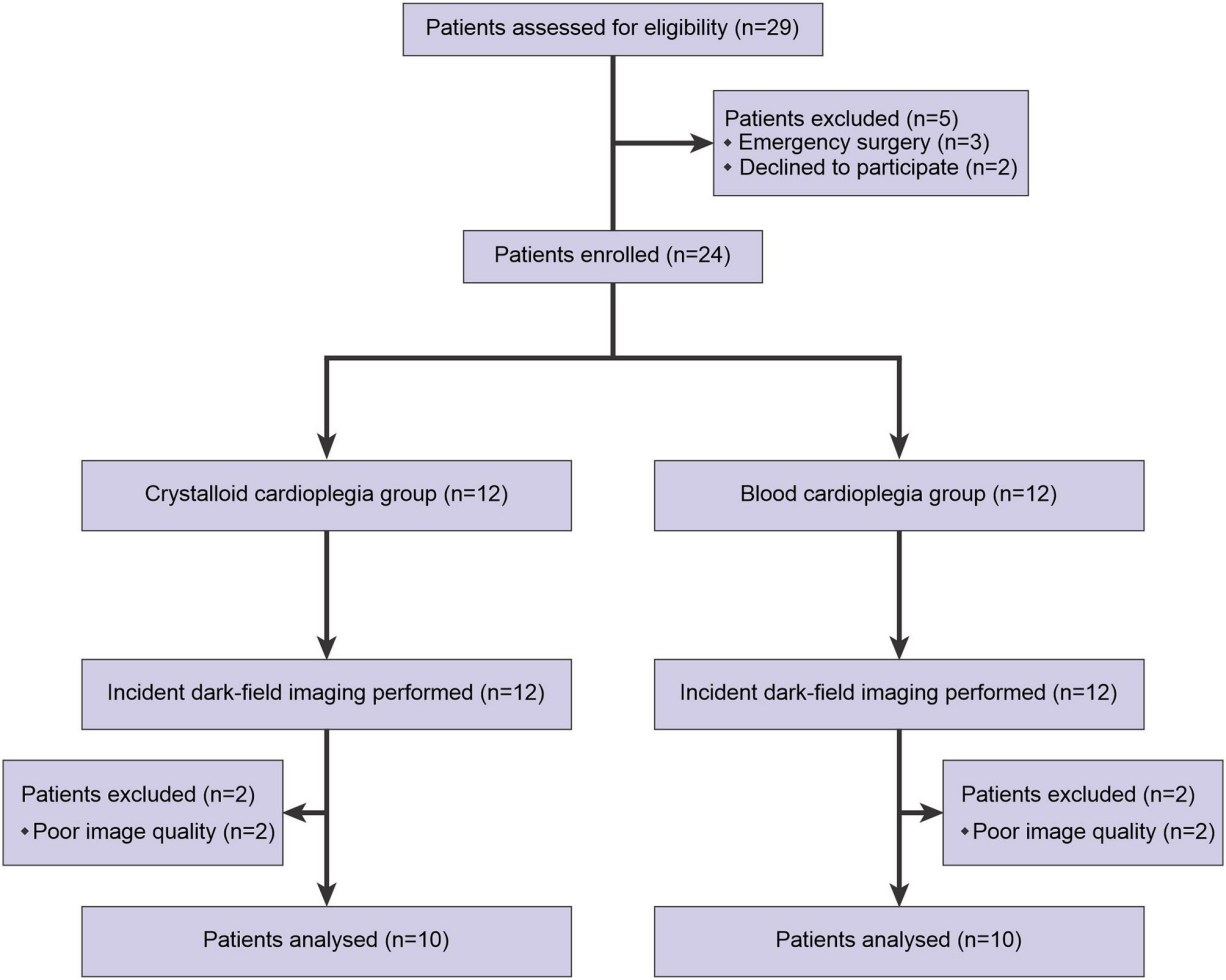
Diagram of recruitment flow to the study.

From 24 patients enrolled in this study, 20 patients assigned for CCG (*n* = 10) or BCG (*n* = 10) had recordings of good or acceptable quality to perform adequate image analysis (Figure 1). The characteristics of these patients are shown in Table 1. No statistically significant differences in demographic, surgery-related, and postoperative outcome parameters were observed between the groups.

**Table 1:** Characteristics of patients.

**Characteristic**	**CCG (*n* = 10)**	**BCG (*n* = 10)**	***p***
**Demographic data**
Age (years)	63.0 ± 3.1	63.9 ± 3.4	0.85
Body surface area (m^2^)	1.9 ± 0.1	2.1 ± 0.1	0.25
EuroSCORE II (%)	0.8 ± 0.1	0.9 ± 0.1	0.95
**Operative details**
Anastomoses (*n*)	2.9 ± 0.2	3.6 ± 0.3	0.06
Surgery duration (minutes)	218.0 ± 12.4	232.5 ± 15.3	0.47
CPB duration (minutes)	69.0 ± 3.5	82.9 ± 6.8	0.09
Cross-clamp duration (minutes)	30 (28.5–39.5)	39.5 (33.8–51)	0.10
**Postoperative follow-up**
Intubation duration (minutes)	528.0 ± 59.9	387.0 ± 34.5	0.06
Stay in intensive care unit (hours)	20.2 ± 0.7	20.0 ± 0.4	0.76
Hospital stay (days)	6.5 (6–8)	6 (6–7.3)	0.44

*Values are presented as mean ± standard error of the mean or median with interquartile range. CCG, Crystalloid Cardioplegia Group; BCG, Blood Cardioplegia Group; EuroSCORE, European System for Cardiac Operative Risk Evaluation; CBP, cardiopulmonary bypass*.

### Intraoperative Microcirculatory Parameters

**Figure 2:**
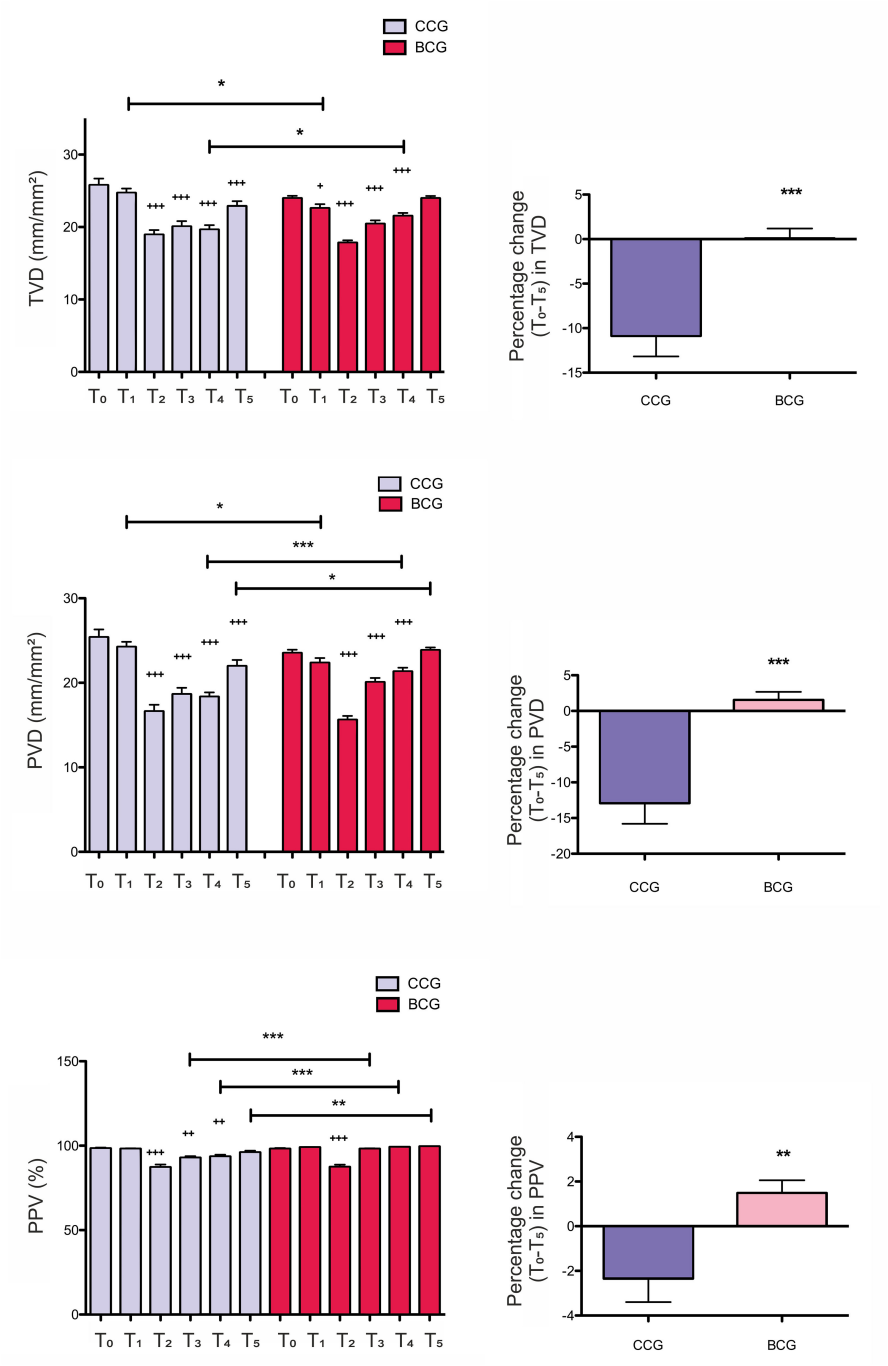
Intraoperative values and related percentage changes of the microcirculatory parameters total vessel density (TVD), perfused vessel density (PVD), and proportion of perfused vessels (PPV) for vessels with a diameter < 20 μm in the crystalloid cardioplegia (CCG) and blood cardioplegia (BCG) groups. All the values are presented as mean ± SEM. T_0_-T_5_, time points; *p < 0.05, **p < 0.01, ***p < 0.001 between groups; ^+^*p* < 0.05, ^++^*p* < 0.01, ^+++^*p* < 0.001 vs. T_0_.

The microcirculatory parameters TVD, PVD, and PPV for vessels with a diameter < 20 μm are shown in Figure 2. At T_1_, TVD in CCG was found to be significantly higher than in BCG before the initiation of ECC [TVD_T1_(CCG) = 24.7 ± 0.62 vs. TVD_T1_(BCG) = 22.58 ± 0.58 mm/mm^2^, *p* < 0.05]. In the both groups, TVD reduced following the initiation of ECC compared with baseline [TVD_T2_(CCG) = 18.93 ± 0.67 vs. TVD_T0_(CCG) = 25.77 ± 0.92 mm/mm^2^, *p* < 0.001; TVD_T2_(BCG) = 17.81 ± 0.36 vs. TVD_T0_(BCG) = 23.96 ± 0.35 mm/mm^2^, *p* < 0.001]. The decline in TVD tended to be temporary in BCG and recovered to baseline levels (Figure 2). At T_4_, TVD in BCG was significantly higher than in CCG [TVD_T4_(CCG) = 19.63 ± 0.63 vs. TVD_T4_(BCG) = 21.51 ± 0.44 mm/mm^2^, *p* < 0.05]. The percentage changes from T_0_ to T_5_ (%Δ)_T0−*T*5_ showed a statistically significant difference between the groups in favor of BCG [%ΔTVD_T0−*T*5_(CCG) = −10.86 ± 2.323 vs. %ΔTVD_T0−*T*5_(BCG) = 0.0804 ± 1.107, *p* < 0.001] (Figure 2).

The effect of the use of different cardioplegia types on PVD is shown in Figure 2. At T_1_, PVD in CCG was significantly higher than in BCG before the onset of ECC [PVD_T1_(CCG) = 24.24 ± 0.61 vs. PVD_T1_(BCG) = 22.35 ± 0.57 mm/mm^2^, *p* < 0.05]. The observed reduction in PVD during ECC in the both groups was only restored in BCG and increased to baseline levels (Figure 2). PVD in BCG was significantly higher than in CCG at time points T_4_ and T_5_ [PVD_T4_(CCG) = 18.33 ± 0.53 vs. PVD_T4_(BCG) = 21.33 ± 0.46 mm/mm^2^, *p* < 0.001; PVD_T5_(CCG) = 21.96 ± 0.75 vs. PVD_T5_(BCG) = 23.85 ± 0.33 mm/mm^2^, *p* < 0.05]. The percentage changes from T_0_ to T_5_ showed a statistically significant difference between the groups in favor of BCG [%ΔPVD_T0−*T*5_(CCG) = −12.91 ± 2.884 vs. %ΔPVD_T0−*T*5_(BCG) = 1.528 ± 1.144, *p* < 0.001] (Figure 2).

The effect of the use of CCG and BCG on PPV is shown in Figure 2. In the both groups, PPV reduced after the initiation of ECC and only recovered in BCG. PPV in BCG was significantly superior than in CCG at time points T_3_, T_4_, and T_5_ [PPV_T3_(CCG) = 92.87 ± 0.96 vs. PPV_T3_(BCG) = 98.15 ± 0.29%, *p* < 0.001; PPV_T4_(CCG) = 93.54 ± 1.1 vs. PPV_T4_(BCG) = 99.12 ± 0.24%, *p* < 0.001; PPV_T5_(CCG) = 96.02 ± 0.99 vs. PPV_T5_(BCG) = 99.5 ± 0.1%, *p* < 0.01]. The percentage changes from T_0_ to T_5_ showed a statistically significant difference between the groups in favor of BCG [%ΔPPV_T0−*T*5_(CCG) = −2.345 ± 1.049 vs. %ΔPPV_T0−*T*5_(BCG) = 1.482 ± 0.576, *p* < 0.01] (Figure 2).

### Intraoperative Hemodynamic and Laboratory Parameters

The intraoperative hemodynamic and laboratory parameters are shown in Table 2. Hematocrit levels decreased after the onset of ECC in the both groups, but remained above the transfusion threshold throughout the CPB course [Hematocrit_T2_(CCG) = 27.1 ± 1.1 vs. Hematocrit_T0_(CCG) = 37.3 ± 1.4%, *p* < 0.01; Hematocrit_T2_(BCG) = 28.4 ± 0.6 vs. Hematocrit_T0_(BCG) = 38.9 ± 0.7%, *p* < 0.01]. According to the intergroup analysis by time points, no statistically significant differences in hematocrit values were found between patients with CCG and BCG before, during, and after the CPB period (Table 2). The body temperature also showed a significant decrease following the initiation of ECC in the both groups [Temperature_T2_(CCG) = 33.1 ± 0.2 vs. Temperature_T0_(CCG) = 35.9 ± 0.1°C, *p* < 0.05; Temperature_T2_(BCG) = 33.5 ± 0.2 vs. Temperature_T0_(BCG) = 36.1 ± 0.1°C, *p* < 0.05]. During CPB, the temperature in CCG was significantly higher than in BCG at T_3_ [Temperature_T3_(CCG) = 32.3 ± 0.2 vs. Temperature_T3_(BCG) = 31.6 ± 0.2°C, *p* < 0.05], but significantly lower at T_4_ [Temperature_T4_(CCG) = 35.0 ± 0.2 vs. Temperature_T4_(BCG) = 35.8 ± 0.3°C, *p* < 0.05]. Remarkably, no correlations were found between the temperature and the microcirculatory parameters throughout the CPB period in either of the study groups. After the termination of CPB, the reduced temperature values recovered to baseline levels without showing any statistically significant differences at T_5_ between patients in CCG and BCG (Table 2). Hemodynamically, all the patients showed a stable course and did not require additional inotropic support within the study period. At time points T_0_ and T_2_, MAP in BCG was significantly higher than in CCG [MAP_T0_(CCG) = 72.4 ± 2.6 vs. MAP_T0_(BCG) = 88.2 ± 4.5 mm Hg, *p* < 0.05; MAP_T2_(CCG) = 44.9 ± 2.1 vs. MAP_T2_(BCG) = 62.3 ± 4.1 mm Hg, *p* < 0.05]. After the onset of CPB, MAP decreased but remained constant between 40 and 80 mm Hg in the both groups. During this period, no correlations were found between MAP and microcirculatory parameters in either of the study groups. After the discontinuation of CPB, MAP values did not reveal any statistically significant differences at T_5_ between patients in CCG and BCG (Table 2). Finally, arterial pH, partial pressure of O_2_, partial pressure of CO_2_, and lactate concentrations did not indicate any differences between the groups during the study period (Table 2).

**Table 2:** Intraoperative hemodynamic and laboratory parameters.

**Variable**	**Group**	**T_**0**_**	**T_**1**_**	**T_**2**_**	**T_**3**_**	**T_**4**_**	**T_**5**_**
**Heart rate (bpm)**	CCG	51.5 ± 3.1	62.9 ± 3.4	64.8 ± 4.0^a^	-	55.6 ± 4.2	73.9 ± 3.8^a^
	BCG	54.5 ± 3.9	62.9 ± 4.7	69.0 ± 5.5^a^	-	64.8 ± 4.8	70.7 ± 3.2
**MAP (mmHg)**	CCG	72.4 ± 2.6	71.6 ± 2.6	44.9 ± 2.1^b^	62.3 ± 4.6^a^	60.8 ± 4.6^a^	66.0 ± 1.5^a^
	BCG	88.2 ± 4.5^d^	78.7 ± 5.6	62.3 ± 4.1^a^, ^d^	60.7 ± 3.9^a^	70.4 ± 4.6^a^	68.8 ± 3.8^b^
**Temperature (** **°** **C)**	CCG	35.9 ± 0.1	35.5 ± 0.1	33.1 ± 0.2^a^	32.3 ± 0.2^a^, ^d^	35.0 ± 0.2^a^, ^d^	36.4 ± 0.1
	BCG	36.1 ± 0.1	35.8 ± 0.1	33.5 ± 0.2^a^	31.6 ± 0.2^a^	35.8 ± 0.3^a^	36.8 ± 0.1
**Haematocrit (%)**	CCG	37.3 ± 1.4	38.3 ± 1.3	27.1 ± 1.1^b^	27.3 ± 1.2^b^	27.6 ± 1.3^b^	29.4 ± 0.9^a^
	BCG	38.9 ± 0.7	39.7 ± 0.9	28.4 ± 0.6^b^	27.0 ± 0.8^b^	29.6 ± 1.2^b^	29.7 ± 0.9^a^
**Arterial pH**	CCG	7.43 ± 0.01	7.38 ± 0.02^c^	7.43 ± 0.01	7.43 ± 0.01	7.45 ± 0.01	7.40 ± 0.02
	BCG	7.42 ± 0.01	7.41 ± 0.01	7.43 ± 0.01	7.42 ± 0.01	7.46 ± 0.02	7.40 ± 0.01
**Arterial pCO**_**2**_ **(mmHg)**	CCG	35.9 ± 1.4	43.0 ± 2.4^c^	40.7 ± 1.6^b^	37.7 ± 1.4	32.6 ± 1.1	37.2 ± 1.6
	BCG	36.9 ± 0.9	39.6 ± 1.4	38.8 ± 1.2	35.4 ± 0.6	32.2 ± 1.0^b^	36.9 ± 1.2
**Arterial pO**_**2**_ **(mmHg)**	CCG	149.6 ± 14.3	160.2 ± 13.6	169.5 ± 14.1	168.3 ± 7.9	194.8 ± 11.0^a^	111.0 ± 8.2
	BCG	156.9 ± 10.8	170.2 ± 14.5	166.9 ± 20.6	187.7 ± 11.0	160.3 ± 16.2	118.0 ± 10.1
**Lactate (mmol/L)**	CCG	0.9 ± 0.1	0.9 ± 0.1	0.9 ± 0.1	1.1 ± 0.1	1.1 ± 0.1	1.3 ± 0.1
	BCG	1.2 ± 0.2	1.0 ± 0.1	1.0 ± 0.1	1.1 ± 0.04	1.4 ± 0.1	1.5 ± 0.1

*Values are presented as mean ± SEM*.

a *p < 0.05 vs. T_0_*.

b *p < 0.01 vs. T_0_*.

c *p < 0.001 vs. T_0_*.

d *p < 0.05 between groups*.

*T_0_-T_5_, time points; CCG, crystalloid cardioplegia; BCG, blood cardioplegia; MAP, mean arterial pressure; pO_2_, partial pressure of oxygen; pCO_2_, partial pressure of carbon dioxide*.

## Discussion

In this study, we evaluated, to the best of our knowledge, for the first time the impact of the use of different cardioplegia solutions on CPB-induced microcirculatory alterations. Our results showed that the use of blood cardioplegia, but not crystalloid cardioplegia resulted in a recovery of microcirculatory changes after the termination of CPB. The courses of systemic hemodynamic and laboratory parameters did not reveal any such differences between the groups after the discontinuation of ECC. With respect to the postoperative outcome, a decrease in the postoperative intubation duration was detected in BCG, which did not reach statistical significance.

Microcirculatory alterations have been shown to occur during cardiac surgery with CPB in several observational studies (7–10), indicating the importance of microcirculatory monitoring in the perioperative care of patients with cardiac surgery. This study primarily contributes to the existing literature by providing first detailed intraoperative measurement of microvascular alterations evaluated *via* IDF imaging, which ensures better image quality and the visualization of more sublingual capillaries than the initially used imaging techniques (12). Our measurements *via* IDF imaging confirm former studies indicating the effects of non-pulsatile CPB on microcirculation (7–10). Indeed, the initiation of ECC was associated with a decreased microvascular density and perfusion in the both study groups. Mechanisms related to these CPB-induced microcirculatory alterations mainly involve hemodilution, systemic inflammatory response, and subsequent endothelial dysfunction (1). Among these, hemodilution due to the addition of the priming volume to the circulating blood results in a dilution of RBCs and, thus, a reduction in hematocrit during CPB. The reduction in hematocrit consequently leads to a decrease in blood viscosity, which can affect microcirculatory perfusion and oxygenation (17). In this study, impaired microcirculatory density and perfusion during hemodilution have been observed in patients of CCG and BCG, indicating the certain impact of CPB initiation in the both groups.

The initiation of CPB was accompanied by a sudden decrease in the microvascular density and perfusion in the both groups. The discontinuation of CPB, however, did not reverse the microcirculatory alterations in patients who received crystalloid cardioplegia during CPB. Sustained inflammatory response and damaged cellular sensing mechanisms may explain the impaired recovery of the microcirculation after the termination of ECC in this cohort (18). Remarkably, the patients who received blood cardioplegia showed a recovery of the microcirculatory changes after the discontinuation of ECC. In this regard, the beneficial effects of the use of blood cardioplegia on the systemic inflammatory response would be in line with our results (5, 6). Basically, the systemic inflammatory response to CPB can be divided into two main phases (19). Among these, the early phase begins immediately after the contact of the blood components with the artificial surface and involves the activation of the innate immune system, resulting in a short-lived response. As this response lessens, a late phase develops, mainly caused by myocardial IRI. During IRI, the heart is exposed to an initial ischemic period associated with the application of the aortic cross-clamping, leading to the production of reactive oxygen species (ROS) (20) as well as cytokines (21). Reperfusion of the heart increases the concentration of these mediators, triggering further activation of the inflammatory pathways. Leukocytes entering the reperfused area, in turn, release cytokines and ROS, which may aggravate the state of inflammation. Following reperfusion, these inflammatory mediators are washed out of the heart to trigger the late phase of the inflammatory response in the systemic (micro)circulation (19). Leukocytes induced by these mediators subsequently transmigrate into the tissues, thereby causing endothelial injury (2). Activated leukocytes likewise release inflammatory mediators which may further harm the microvascular endothelium (22). The resultant inability of microvascular endothelial cells to constitutively produce nitric oxide (22), in turn, affects the balance between vasoactive substances in favor of vasoconstriction (23), which is aggravated by the prothrombotic state associated with endothelial dysfunction (24). As a result, microcirculatory alterations may occur, causing inadequate tissue perfusion and oxygenation. However, in case of the use of blood cardioplegia, myocardial IRI can be reduced in patients undergoing ECC conditions (25), leading to a reduction of inflammatory mediators released into the systemic (micro)circulation. The resultant attenuation of the late phase of the inflammatory response may then prevent the microcirculatory injury during this period, enabling a recovery of the “early-phase” microcirculatory alterations, as reflected by the increase in microcirculatory parameters in BCG patients in this study.

Indeed, endothelial dysfunction can also be caused by direct effects of cardioplegic solutions on the endothelium, which, can, in turn, induce an inflammatory response (26). A well-known mechanism of endothelial damage related to crystalloid cardioplegic solution is the effect of low osmotic pressure on the endothelial surface (26). In this regard, increasing the osmotic pressure of cardioplegic solution has been shown to ameliorate the endothelial injury (27). Similarly, glucose added to blood cardioplegia solution to increase its osmolality may also play a beneficial role in lessening the microvascular endothelial damage in this cohort.

Significantly, the systemic hemodynamic and laboratory parameters were not associated with the recovery of microcirculatory parameters in BCG patients, since the courses of these variables did not reveal differences between the study groups after the termination of CPB. In addition, no significant correlations were found between hemodynamic measures, particularly MAP and microcirculatory parameters throughout the CPB course in either of the study groups, confirming the minor role of systemic circulation in the resuscitation of microcirculation during cardiac surgery (1).

A limitation of this study concerns the small number of patients, making it difficult to generalize our findings. Indeed, this study demonstrates that the use of blood cardioplegia significantly ameliorated the microcirculatory changes that were induced by CPB. But, future randomized trials are needed to confirm these findings. Regrettably, we also did not investigate the underlying mechanisms that may explain the beneficial effects of blood cardioplegia on microcirculation. Moreover, a low-risk patient population, which underwent uncomplicated cardiac surgery, was included in this study. We expect that further studies with larger and high-risk sample sizes will overcome these shortcomings.

## Conclusion

The use of blood cardioplegia ameliorates CPB-induced microcirculatory alterations better than crystalloid cardioplegia in patients undergoing CABG, which may reflect attenuation of the systemic inflammatory response. Future investigations with larger and high-risk sample sizes and longer follow-up may enable to differentiate the underlying mechanisms of the beneficial effects of blood cardioplegia on microcirculation. Additionally, it remains to be clarified whether the recovery of microcirculatory alterations due to the use of blood cardioplegia is associated with an improved outcome in a high-risk patient population.

## Data Availability Statement

The data that support the findings of this study are available from the corresponding author upon reasonable request.

## Ethics Statement

This study was approved by the Ethics Committee of the School of Medicine at Acibadem Mehmet Ali Aydinlar University (ATADEK 2013-540). All the procedures that were followed in the study were in accordance with the 1964 Helsinki Declaration and its later amendments. Informed written consent was obtained from all participants.

## Author Contributions

GA participated in the design of the study, contributed to the data acquisition, performed the image analysis, and drafted and revised the manuscript. CI participated in the design of the study, contributed to the manuscript revision, and is the guarantor of this study. HU and UA performed the statistical analysis and participated in the manuscript revision. SA performed the image analysis and revised the manuscript. FT, HK, and CA contributed to the coordination of this study and participated in the manuscript revision. All authors read and approved the final version of the manuscript.

## Conflict of Interest

CI has developed SDF imaging and is listed as an inventor on related patents that were commercialized by MicroVision Medical (MVM) under a license from the Academic Medical Center (AMC). He receives no royalties or benefits from this license. He has been a consultant for MVM in the past, but has not been involved with this company for more than 5 years and holds no shares or stock. Braedius Medical, which is a company that is owned by a relative of CI, has developed and designed a handheld microscope, namely, the CytoCam-IDF imaging microscope. The images used in this study were obtained using this technology. CI has no financial relationship with Braedius Medical of any sort. He has never owned shares or received consultancy or speaker fees from this company. Active Medical BV Leiden, a company founded by CI, runs an internet site (http://microcirculationacademy.org), which offers educational courses and services that are related to clinical microcirculation. CI discloses that he is a shareholder of this company. The remaining authors declare that the research was conducted in the absence of any commercial or financial relationships that could be construed as a potential conflict of interest.

## Publisher's Note

All claims expressed in this article are solely those of the authors and do not necessarily represent those of their affiliated organizations, or those of the publisher, the editors and the reviewers. Any product that may be evaluated in this article, or claim that may be made by its manufacturer, is not guaranteed or endorsed by the publisher.
